# Mucin induces CRISPR-Cas defense in an opportunistic pathogen

**DOI:** 10.1038/s41467-022-31330-3

**Published:** 2022-06-25

**Authors:** Gabriel Magno de Freitas Almeida, Ville Hoikkala, Janne Ravantti, Noora Rantanen, Lotta-Riina Sundberg

**Affiliations:** 1 University of Jyväskylä, Department of Biological and Environmental Science and Nanoscience Center, Jyväskylä, Finland; 2 Faculty of Biosciences, Fisheries and Economics, Norwegian College of Fishery Science, UiT The Arctic University of Norway, Tromsø, Norway; 3 University of Helsinki, Molecular and Integrative Biosciences Research Programme, Helsinki, Finland

**Keywords:** Microbial ecology, Bacteriophages, CRISPR-Cas systems

## Abstract

Parasitism by bacteriophages has led to the evolution of a variety of defense mechanisms in their host bacteria. However, it is unclear what factors lead to specific defenses being deployed upon phage infection. To explore this question, we co-evolved the bacterial fish pathogen *Flavobacterium columnare* and its virulent phage V156 in presence and absence of a eukaryotic host signal (mucin) for sixteen weeks. The presence of mucin leads to a dramatic increase in CRISPR spacer acquisition, especially in low nutrient conditions where over 60% of colonies obtain at least one new spacer. Additionally, we show that the presence of a competitor bacterium further increases CRISPR spacer acquisition in *F. columnare*. These results suggest that ecological factors are important in determining defense strategies against phages, and that the phage-bacterium interactions on mucosal surfaces may select for the diversification of bacterial immune systems.

## Abstract (web summary)

It is unknown what circumstances promote particular bacterial defenses against bacterial viruses (phages). Almeida & Hoikkala et al. show that mucin, derived from mucus, greatly accelerates CRISPR-Cas defenses against phage in an opportunistic pathogen.

## Introduction

One of the central themes in host-parasite interactions is to understand the diversity and ecology of host defense strategies^1–4^. Variation in defenses increase plasticity: one defense strategy may be useful in one setting but inefficient or costly in another^1^. However, the ecological conditions that lead to the selective deployment of different immune strategies are still poorly understood. Defense mechanisms may be especially complex in systems where multiple trophic levels are involved^5^. For example, pathogenic bacteria that infect eukaryotic hosts are also parasitized by their viruses, bacteriophages (phages). This tripartite cross-domain relationship involves two layers of interactions that are influenced by host defenses: one between the bacterial host and the phage, and another between the metazoan host and the pathogenic bacterium. The interplay of these layers is still poorly understood^6^, and the continuum of evolutionary interactions extends to even those between the phage and the metazoan^7–12^.

Traditionally, phage-bacterium interactions are studied in simplified laboratory conditions. However, in real life, the interactions between pathogenic bacteria and phages often occur on the mucosal surfaces of vertebrate hosts. Phages are important members of mucosal microbiomes^13^, and some have evolved a symbiotic relationship with eukaryotes by interacting with mucin glycoproteins on the mucosal surfaces^8^. This phage-metazoan mutualism enhances phages’ probability of encounter with bacterial hosts while providing the host external mucosal immunity against invading bacteria^7–9^. Bacterial invasion into mucosa thus leads to competition for space, and resources, and more importantly, subjects bacteria for potential phage infections. Interaction with the mucosa directly increases the virulence of many bacterial pathogens^7,14,15^. Recently, virulence upregulation by mucin exposure was correlated to increased susceptibility to phage infections in *Flavobacterium columnare* and *Aeromonas sp*^7^. Increased phage susceptibility in the mucosal environment might also play a role in the recent descriptions of a *Clostridium difficile* phage activity being improved by eukaryotic cells^11^ and the mucin enhancement of an *Escherichia coli* phage^10^. How the mucosal environment influences bacterial resistance against phages is so far unknown. If defenses against phages incur tradeoffs in virulence for pathogenic bacterial species, conflicts may emerge. Thus, defense strategies that minimize virulence trade-offs during colonization may be favored.

Bacterial defense mechanisms against phage infections are numerous and have evolved towards almost all phases of phage life cycles^16^. Surface modification (SM) is an extracellular defense response, in which the mutation, downregulation or deletion of cell surface proteins prevents phages from attaching to the cell^16^. Within the cell, restriction-modification and CRISPR-Cas systems recognize invading phage genomes by distinguishing them from host nucleic acids and targeting them for destruction. CRISPR-Cas is an adaptive immune system that stores genetic memories of phage genomes (spacers) into a repeat-spacer array in a CRISPR locus. Upon a subsequent infection, the array is expressed to CRISPR-RNA (crRNA)^17^. The resulting repeat-spacer oligos guide endonucleases, such as Cas9, to the spacer-complementary sequence in the phage genome that is then cut by the endonuclease^18,19^.

Since phage receptor proteins often play an important role in bacterial nutrient intake, secretion, motility, or virulence, disruptions in these proteins generally impose fitness costs^16,20,21^. It has been suggested that these tradeoffs are important for maintaining phage-bacterium coexistence^21^. Costs may be permanent or short-lived: constitutive defenses, such as SM, impose continuous costs, whereas inducible defenses, such as CRISPR-Cas, minimize their costs by being generally activated only under specific stimuli. The investment in inducible or constitutive defenses depends on their relative costs in a given environment^3^. For example, surface receptors required for virulence can have major fitness costs if they are mutated during host colonization^22^.

Despite the sophistication of CRISPR-Cas, less than half of all known bacterial species carry CRISPR-Cas loci^23^, implying they may induce fitness costs or be useful only in limited circumstances. Ecological variables such as temperature and oxygen levels are indeed important factors that correlate with CRISPR-Cas occurrence^24^. Interestingly, type II CRISPR-Cas systems seem to be enriched in pathogenic bacteria^24^, and CRISPR-Cas has been suggested to regulate bacterial virulence in some bacterial species^25^, e.g. in *Francisella novicida*^26^ and *Campylobacter jejuni*^27,28^. However, as multiple defenses may be beneficial^1^, bacteria with CRISPR-Cas do not rely exclusively on this defense. The relative costs of CRISPR-Cas have been found to increase with phage concentration, so that above a certain threshold, these costs exceed those of SM, leading to a shift in defense strategy^3^. Furthermore, the presence of competing bacteria can select for increased CRISPR-Cas-based resistance due to an amplification in SM-based costs^29^ The factors that influence defense strategies are still largely unknown and they may have important effects on phage evolution (e.g. via genomic mutations or the evolution of anti-CRISPR proteins) and population size.

We hypothesize that the mucosal interface may be a tipping point between CRISPR-Cas and SM defenses. To investigate this idea we used the opportunistic fish pathogen *F. columnare* that causes columnaris disease in freshwater fish^30^. This bacterium has type II-C and VI-B CRISPR-Cas loci, both active in natural and laboratory environments^31,32^. Exposure to the high concentration of phage in laboratory conditions elicits SM defense in *F. columnare*, causing loss of virulence and motility associated with colony morphotype change from Rhizoid to Rough^33^. Low phage pressure and low nutrient level, on the other hand, can be used to trigger CRISPR-Cas spacer acquisition^32^. Exposure to primary catfish mucus has been shown to upregulate several virulence genes, as well as the CRISPR-Cas adaptation gene *cas2*^34^.

How simultaneous exposure to host signals (mucin), competing bacteria, and phage affects the choice of phage defense strategy between CRISPR-Cas and SM has not been previously explored, despite the mucosal environment being a hotspot for microbial interactions. We reveal that low-nutrient and simulated mucosal environments influence the ecology of phage resistance, in co-cultures of *F. columnare* strain B245 and its virulent phage V156. We also evaluate the effect of bacterial competition (with *Aeromonas sp*. bacteria) for phage resistance in the simulated mucosal condition. We show that while SM is a central resistance mechanism for bacteria, mucosal environment and bacterial competition significantly increases CRISPR spacer uptake. CRISPR-Cas activation by mucin exposure could allow the bacterial pathogen to remain virulent while evading predation by mucosal phages. We also show that SM-mutants are poor competitors compared to the wild-type form in the absence of phage. These results provide crucial information on the ecological factors that shape immune defense strategies that are relevant for bacterial colonization of a vertebrate host.

## Results

### Presence of mucin stabilizes survival of both the bacterium and the phage during 16 weeks of co-culture

**Fig. 1:**
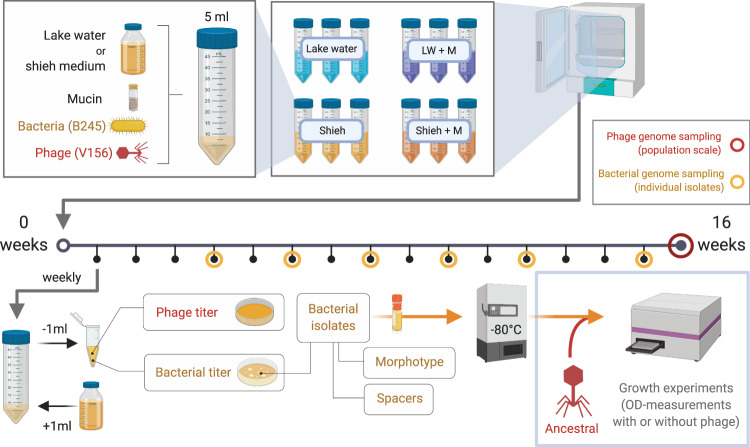
Overview of the experimental setup. The 16-week experiment (denoted by the horizontal line) contained four culturing conditions, which were sampled and restocked with fresh media weekly. Bacterial isolates were characterized by their morphotype and CRISPR spacer content and used later for growth tests with or without the ancestral phage. Phage genomes (population level) were sequenced at week 16. Genomes from representative bacterial isolates were sequenced throughout the experiment. Figure made in ©BioRender - biorender.com.

**Fig. 2:**
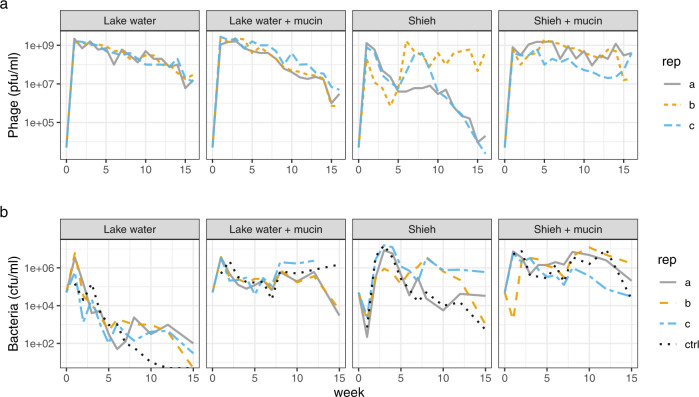
Phage V156 (a) and *F. columnare* B245 (b) titers over the 16-week experiment in the four treatments. Each line represents one of the three replicates in each treatment. The dotted lines in (**b**) are control cultures without phage. Source data are provided as a Source Data file.

An overview of our main experimental setup is shown in Fig. 1. To avoid population bottlenecks, our sampling was based on the weekly collecting 20% of the cultures and replacing with the same volume of fresh medium. Long term co-existence of both *F. columnare* B245 and its phage V156 was observed in all treatments. In lake water with (LW + M) or without mucin (LW), the closest approximations of natural conditions for *F. columnare*, the phage titers remained similar until week 9, after which LW + M showed a significant decline in phage numbers compared to LW (LM, *t*_1,46_ = −2.737, *P* = 0.0088) with roughly a ten-fold difference at week 16 (Fig. 2a, Supplementary Fig. 1a). Bacterial population densities in these treatments were opposite and more dramatic, with an average of 45-fold higher numbers in LW + M than in LM across all time points after an initial spike at week 1 (LM, *t*_1,77_ = 4.836, *P* < 0.001) (Fig. 2b, Supplementary Fig. 1b). Surprisingly, bacteria in the no-phage control of LW became extinct after week 10, while no extinction occurred in the phage-containing cultures.

Cultures in Shieh medium (without mucin) had large variations between replicates in both bacterial and phage titers. Despite similar bacterial titers at week one, differences between replicates grew to more than 100-fold towards the end of the experiment (Fig. 2b). Phage titers declined in Shieh for the first 4–5 weeks, after which replicates *b* and *c* recovered while replicate *a* stabilized. Around week 10, phage titers declined sharply in replicates *a* and replicate *c*, while replicate *b* remained with high titer until the end (Fig. 2a). The presence of mucin in Shieh decreased variation between replicates compared to Shieh alone, with higher phage and bacterial titers (Fig. 2). No statistical analysis was performed on the Shieh cultures due to high divergence in replicates.

### Presence of mucin enhances spacer acquisition in type II-C and VI-B CRISPR-Cas loci

**Fig. 3:**
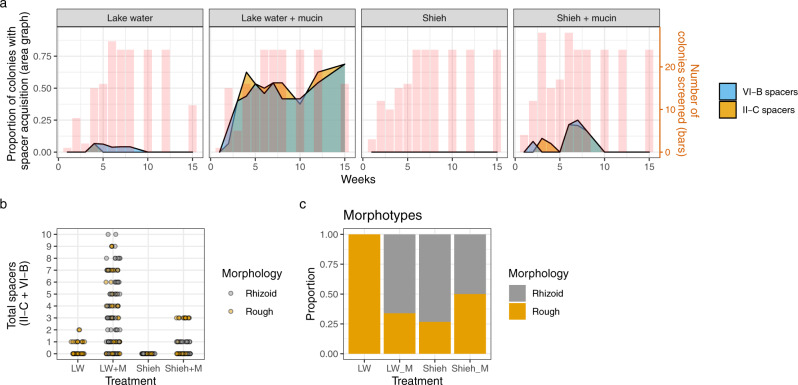
Dynamics of CRISPR spacers and colony morphotypes. **a** CRISPR-Cas spacer acquisition over time in both loci. The area graphs (left Y-axis) represents the proportion of colonies in which the CRISPR-Cas array expanded by at least one new spacer. Values are means from the three replicates. The red columns (right Y-axis) show the total number of colonies that were screened at each time point to obtain these proportions. Missing red bars indicate no screening on that week – the respective proportional data is, therefore, an interpolation. **b** The absolute number of spacers from individual isolates in different treatments. New spacers in both loci have been added together. **c** Morphotype distribution across the treatments (isolates from all time points pooled together). Source data are provided as a Source Data file.

Culture conditions, especially the presence of mucin, had a significant impact in the acquisition of new CRISPR spacers (Fig. 3a, b). The presence of mucin in lake water increased spacer acquisition 9-fold compared to plain lake water (GLMM, *Z* = 4.271, *P* < 0.001), and the efficiency of the acquisition was 5-fold when comparing lake water with mucin to Shieh with mucin (GLMM, *Z* = −3.367, *P* < 0.001) (Fig. 3b). Timewise, the maximum efficiency of the acquisition was reached around week 3 in the LW + M treatment, with over 60% of LW + M colonies having acquired new spacers (Fig. 3a). The number of spacers in one locus in a single isolate was up to six spacers in LW + M, two in LW and up to three in Shieh with mucin (Fig. 3b). The efficiency of spacer acquisition was roughly similar between the two CRISPR loci regardless of the treatment. Shieh without mucin did not show any new spacers.

Surprisingly, spacer acquisition was not exclusive to the Rhizoid morphotype. In fact, in Shieh + M treatment, isolates with the highest number of spacers were Rough, indicating an overlap of surface modification and spacer acquisition (Fig. 3b). However, after a peak in isolates with CRISPR spacers in this treatment around week 7, no more spacer mutants appeared. When only considering morphotype, LW had no Rhizoid colonies while other conditions had a minor bias towards them (Fig. 3c).

### Co-culturing with phage leads to immunity

**Fig. 4:**
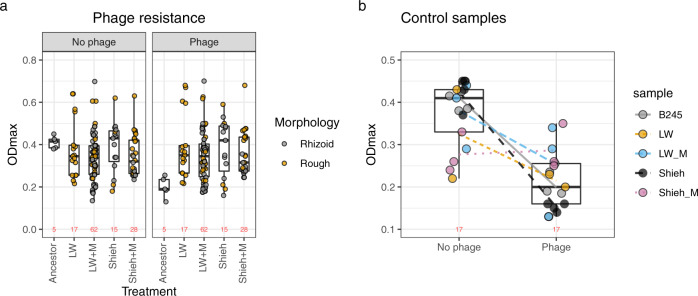
Effect of environment and colony morphology on bacterial growth. Each dot represents the mean of three replicate OD measurements of an isolate. The box plots capture the minimum and maximum values, 25th and 75th percentile (box) and the median (black horizontal line) of these dots. The number of observations is shown in red for each dataset. **a** Ancestral B245 and isolates originating from the four treatments in the presence or absence of phage. **b** Control samples (grown without phage) from the same four treatments plus the ancestor in the presence or absence of phage. The box plots represent all treatments, including ancestor bacteria. Lines visualize the change in mean ODmax per sample. Source data are provided as a Source Data file.

To detect the development of phage resistance and any associated costs during the 16-week co-culture experiment, we grew bacterial isolates obtained during the experiment in the presence or absence of the ancestral phage and compared the maximum OD (OD_MAX_) reached to that of ancestral B245. In principle Rhizoid colonies are phage susceptible while Rough colonies are phage resistant due to SM. In the absence of phage, the OD_MAX_ of the four treatments (LW, LW + M, Shieh, Shieh+M) did not significantly differ from the ancestral B245 (Fig. 4a). In the presence of phage, however, the OD_MAX_ of the ancestral B245 decreased from 0.41 to 0.19 (GLMM, *Z* = −13.78, *P* < 0.001), while OD_MAX_ in the four treatments remained unchanged, indicating phage resistance (GLMM, *Z* = −0.281 to −0.599, *P* > 0.3 in all) (Fig. 4a). These results suggest that co-culturing *F. columnare* with phage caused phage resistance to evolve in all four treatments.

We also measured the growth of isolates from the control cultures from the 16-week experiment (same conditions but without phage). As expected, isolates from most treatments had lower OD_MAX_ in the presence of phage compared to the absence of phage (Fig. 4b). The only exception was the Shieh + M isolate, which had a significantly lower OD_MAX_ in the absence of phage compared to the ancestral bacterium (OD 0.41) with a predicted OD of 0.276 (LM, *t*_2,24_ = −3.053, *P* = 0.0055). In the presence of phage, however, this control had a predicted OD of 0.287, which is significantly higher than that of the ancestor’s 0.199 (LM, *T*_2,24_ = 3.565, *P* = 0.0016). These results suggest that prolonged incubation in Shieh + M in the absence of phage made the cells grow slower, but also made them phage resistant, perhaps incidentally through adaptation to the nutrient-rich mucin environment.

### Fitness benefits of CRISPR adaptation depends on the environment

**Fig. 5:**
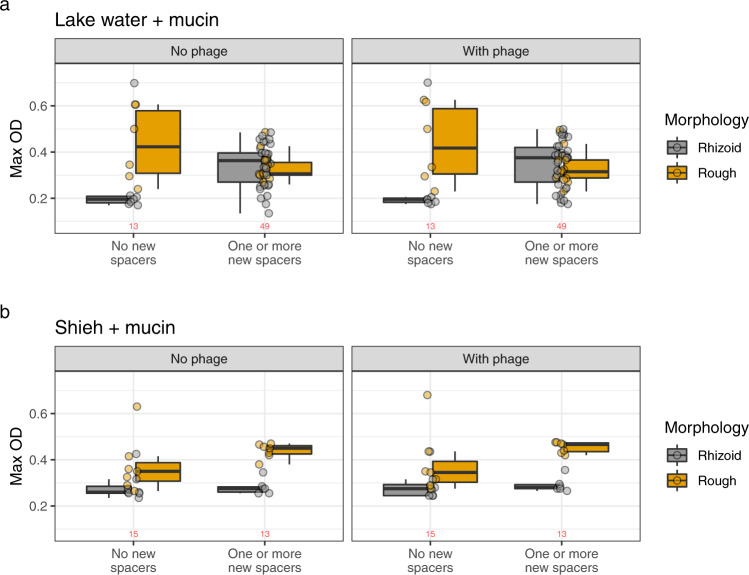
The effect of spacers and morphotype in LW + M and Shieh + M isolates on bacterial growth. Isolates are grouped into having no new spacers or having one or more new spacers regardless of the CRISPR-Cas locus. Dots represent individual observations. The box plots capture the minimum and maximum values, 25th and 75th percentile (box) and the median (black horizontal line). The number of observations is shown in red for each dataset. **a** Isolates from Lake water + mucin treatments. **b** Isolates from Shieh + mucin treatments. Source data are provided as a Source Data file.

We next investigated how spacer acquisition in different morphotypes affected bacterial growth. This analysis was only done for the LW + M and Shieh+M treatments, which produced enough isolates with expanded CRISPR arrays for statistical analysis. In the absence of phage, Rhizoid and Rough isolates with native CRISPR arrays from the LW + M treatment reached predicted OD_MAX_ of 0.173 and 0.421, respectively (Fig. 5a). This difference was statistically significant (GLMM, *Z* = 6.34, *P* < 0.001). However, the acquisition of one or more spacers almost doubled the predicted OD_MAX_ of Rhizoid isolates from 0.173 to 0.337 (GLMM, *Z* = 5.56, *P* < 0.001). The effect of new spacers was opposite for rough isolates, whose OD_MAX_ was reduced by 20% to a predicted 0.340 (GLMM, *Z* = −5.326, *P* < 0.001). These effects were similar and significant also in the presence of phage (Fig. 5). Sanger sequencing of selected bacterial isolates revealed that all sequenced spacers in the type II-C CRISPR-Cas locus were targeting the phage (16/16), while in the VI-B locus roughly two-thirds (19/28) were targeting the phage and the rest (9/28) targeting the bacterial genome (Supplementary data 1).

In Shieh+M samples without phage, Rough isolates had a significantly higher OD_MAX_ than Rhizoid ones (0.400 vs 0.339, GLMM, *Z* = 2.240, *P* = 0.025) (Fig. 5b). Surprisingly, new spacers increased OD_MAX_ of Rough isolates by 40% (GLMM, *Z* = 2.589, *P* = 0.01) but did not affect Rhizoid OD_MAX_. Again, results were similar when these isolates were subjected to phage (Fig. 5b).

Overall, CRISPR spacer acquisition has a positive impact on Rhizoid colonies and a negative impact on Rough colonies when the isolates originate from an environment that favors spacer acquisition (LW + M, Fig. 1a). However, when isolates originate from a nutrient-rich environment that does not favor CRISPR-Cas (Shieh + M, Fig. 1a), there is no or little benefit from the acquired spacers.

### Competition in lake water supplemented with mucin enhances spacer acquisition

**Fig. 6:**
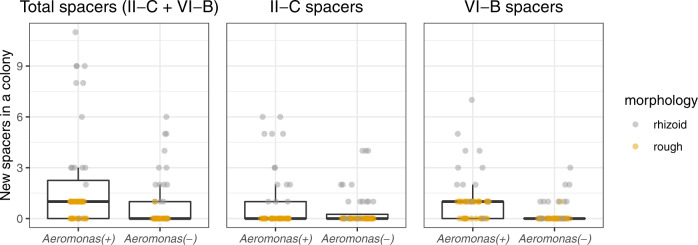
Effect of competitor on CRISPR-Cas spacer acquisition. The Y-axis shows the total number of spacers in a single *F. columnare* colony in the presence or absence of *Aeromonas sp*. The box plots capture the minimum and maximum values, 25th and 75th percentile (box) and the median (black horizontal line). Source data are provided as a Source Data file.

After detecting that mucin in lake water caused an increase in CRISPR spacer acquisition, we performed a follow-up experiment testing the effect of competing bacterial species on *F. columnare* spacer acquisition in this condition. Our aim was to see if the presence of a competing bacterial species would provoke *F*. columnare to maintain phage defenses that enable efficient nutrient intake (CRISPR-Cas) in a competitive setting. Initially, we chose *Aeromonas sp*. which responds to mucin similarly as *F. columnare* by forming biofilm and by becoming more susceptible to phage infections, and *E. coli* DSM613 for apparently not being affected by mucin exposure^7^. However, the *E. coli* populations were either extinguished quickly (no colonies were seen in the first samplings) or remained at very low levels (a few colonies were seen at the last time point) during the experiment (Supplementary fig. 2). This, allied to the fact that we could not measure which effect the initial input of *E. coli* had to mucin content in the cultures, led us to discard the *E. coli* -containing conditions from the final analysis. The presence of *Aeromonas sp*. significantly enhanced *F. columnare* spacer acquisition as measured by the total number of spacers acquired across CRISPR loci (Fig. 6). In the absence of *Aeromonas sp*., the expected total number of new spacers in *F. columnare* was 0.57 per colony, whereas in a co-culture each colony was expected to acquire 1.64 spacers (GLMM, *Z* = −3.381, *P* < 0.001). Interestingly, in this experiment the Rhizoid colony type bacteria acquired more spacers than the Rough types (Fig. 6).

**Fig. 7:**
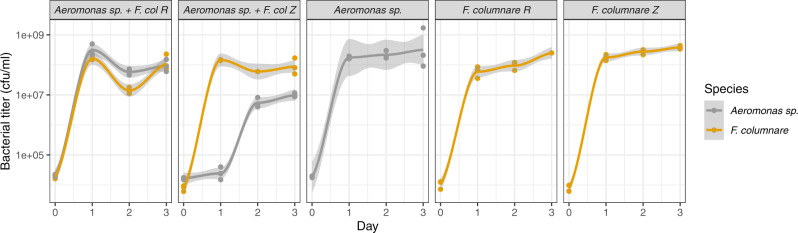
Competition experiment with *Aeromonas sp*. and Rhizoid (Z) and Rough (R) *F. columnare* in lake water + mucin. Y-axis shows mean bacterial cell numbers of three replicate cultures. Plots smoothed using the loess function in R, with grey areas indicating 95% confidence interval across the replicates. Source data are provided as a Source Data file.

Given the association of Rhizoid colony type with spacer acquisition in this competitive environment, we tested if morphotype affects *F. columnare* competetiveness. To test this, we grew *Aeromonas sp*. in the presence of either Rhizoid or Rough *F. columnare*. As controls, both species were grown alone. The presence of Rhizoid *F. columnare* dramatically reduced the concentration of *Aeromonas sp*. in all time points compared to the *Aeromonas sp*. control (GLM, t_2,23_ = −8.185, *P* < 0.001), whereas rough *F. columnare* did not have a significant effect on *Aeromonas* (GLM, *t*_2,23_ = −1.580, *P* = 0.128) (Fig. 7).

### Genome analysis

We sequenced phage and bacterial genomes to search for genetic variations resulting from coevolution. We also sequenced the control bacterial genomes (evolved without phage) to differentiate between mutations caused by interaction with phage and those that may arise from different culturing conditions.

Phage genomes were investigated on the population level: at the end of the 16-week experiment, phage samples of each culture (representing the variety of phages present in that culture) was used to infect the ancestral bacterium and the resulting lysate was deep sequenced. We found an abundance of shared mutations across multiple replicates. Due to the low likelihood of the same mutations occurring convergently across multiple samples (suggesting common sequencing artefacts), we discarded most mutations as false positives (however, all mutations are listed in Supplementary data 2). We were confidently able to recover individual mutations in only two cultures: Replicate *c* from Shieh + M treatment had a non-synonymous mutation (A_357_T) in a predicted phage baseplate protein and a nonsynonymous mutation (M_200_V) in a putative protein with no predicted function (Table 1). Replicate *b* from Shieh treatment had a non-synonymous mutation in a protein with an unknown function near the aforementioned baseplate protein (V_300_I), as well as a synonymous mutation (K_10_K) in a predicted DNA helicase (Table 1). Common to both replicates is that they showed a drop in phage titer after the initial spike but recovered towards the end (Fig. 2a). In fact, replicate *b* in Shieh treatment was the only replicate in its treatment group in which the phage titer remained high at the end of the experiment.

**Table 1:** Mutations discovered in phage genomes on the population level.

Treatment	Replicate	Frequency	Position	AA change	Protein prediction
Shieh + mucin	*c*	11.3%	22,215	A_357_T	Baseplate wedge protein
Shieh + mucin	*c*	6.6%	38,183	M_200_V	Unknown
Shieh	*b*	19.4%	24,026	V_300_I	Unknown*
Shieh	*b*	6.9%	40,807	K_10_K	DNA helicase

The frequency column shows the percentage of reads the mutation was found in. Position refers to the B245 genome. *While the function of this protein could not be predicted, its genomic neighborhood suggests a role as a phage structural protein.

Bacterial genomes were investigated on the isolate (clonal) level. Isolates were picked from different treatments at several time points during the experiment. Evidence of surface modification was found in almost all phage-exposed samples in the flavobacterial gliding motility genes which are associated with type IX secretion system and whose mutations are expected to cause colony morphotype change from Rhizoid to Rough^35^. Most of these mutations caused premature stop codons or introduced frameshift mutations (Table 2). Most of the T9SS mutants were Rough (Table 2). Isolates from the control treatments without phage did not show mutations in gliding motility genes but had variation in other ORFs. It is therefore possible that extended growth in these conditions introduced other adaptive changes, although these mutations were not present in the phage-exposed samples. Indeed, most variation was detected in the Shieh + M control isolate, which seemed to have had developed resistance against phage even in the phage’s absence (Fig. 2b). This sample contained non-synonymous mutations in several metabolism related genes (e.g. Lon, rpoB and surE), and in a putative type VI secretion system-like gene (Supplementary data 3). Type VI secretion systems have previously been shown to be associated with host colonization and bacterial antagonism in *Flavobacterium johnsoniae*^36^. Mutations in the *gld* and *spr* genes resulted in resistance against the ancestral V156 phage.

**Table 2:** Bacterial mutations in gliding motility-related genes.

Treatment (replicate)	Week	Colony morphotype	Gliding motility mutation	Mutation type	New spacers (IIC-VIB)	Resistance
LW (b)	4	Rough	*sprA*	Stop codon	0–0	R
LW (b)	4	Rough	*sprA*	Stop codon	0–2	R
LW (b)	8	Rough	*sprA*	Stop codon	0–1	R
LW (c)	15	Rough	*gldM*	Stop codon	0-0	R
LW + M (a)	15	Rough	*gldK*	SNP nonsynonymous	0–0	R
LW + M (b)	12	Rhizoid	*gldK* (intergenic)	Intergenic poly-AT	2–1	R
LW + M (b)	15	Rhizoid	*sprA*	Single nt deletion -> frameshift middle of gene	0–0	R
LW + M (c)	8	Rhizoid	*none*		3–4	10^−2^
Shieh (a)	12	Rough	*sprA*, *SusC/RagA TonB*-linked outer membrane protein	*sprA*: poly-A 8->7. *SusC*: 48 bp deletion	0–0	R
Shieh (b)	8	Rough	*none*		0–0	R
Shieh (c)	10	Rhizoid	*none*		0–0	10^−3^
Shieh + M (a)	12	Rhizoid	*ompA*	SNP nonsynonymous	0–0	10^−3^
Shieh + M (a)	8	Rough	*ompA*, *sprE*	*ompA*: (CCATCA)repeat 3->2. *sprE*: poly-A 7->8	1–2	R
Shieh + M (c)	8	Rough	GldN, gliding motility-related gene (LOCUS_17550)	GldN: 1 bp deletion -> frameshift. LOCUS 17550: 42 bp deletion	0–0	R
LW (ctrl)	3	Rhizoid	*none*		0–0	10^−2^
LW + M (ctrl)	15	Rough	*none*		0–0	10^−5^
Shieh (ctrl)	12	Rough	*none*		0–0	10^−5^
Shieh + M (ctrl)	15	Rhizoid	Putative T6SS -related gene		0–0	10^−5^
Ancestor B245		Rhizoid			0–0	10^−5^

For a detailed list of all mutations, see Supplementary data 2. Resistance: R indicates complete phage resistance, and numbers indicate the last phage dilution that caused observable plaques in the bacterial lawn (phage V156 stock concentration 10^10^ pfu mL^−1^).

## Discussion

Environmentally transmitted opportunistic bacteria survive long periods in the environment, which may select for reduced growth and metabolic rate^37^. Whereas the interactions between pathogenic bacteria and their phages are generally studied in well-defined laboratory conditions, in real life they often interact in the complex mucosal surfaces of the vertebrate host. Despite the central role of mucosal surfaces for health, there are several fundamental gaps in our knowledge regarding the biology of the phage-bacterium interactions in the mucosal environment. Chemical signals of the host regulates bacterial genes needed for invasion and virulence^34^, generating a physiological state exploited by mucosal phages for infection^7^. Moreover, the ability to colonize hosts is often associated with trade-offs in phage resistance^22^. It is therefore relevant to ask how the mucosal environment affects relative investment into different bacterial defense mechanisms against phages. Here, we investigated this question using the opportunistic pathogen *F. columnare* that causes mucosal disease in freshwater fish^30^ but is known to be resilient and able to withstand adverse conditions outside the host^37,38^. Our study suggests that mucin has a central role in triggering CRISPR-Cas immunity and in buffering bacterial survival.

The lake water conditions with and without mucin (LW + M, LW) used in this study are the closest approximations of natural conditions for *F. columnare* thus far. The starved bacterial population (LW) relied almost completely on extracellular immunity via surface modifications, leading to steady decline in the bacterial population density over the 16-week experiment. As the control isolate went extinct in the LW treatment but phage-exposed replicates did not, it is possible that phages have a positive effect on the long-term survival of the bacteria population during starvation, perhaps through resistant cells feeding on lysed ones or through Cas13-induced dormancy by the VI-B CRISPR-Cas locus^39^. However, this result could be a fluctuation in our experiment due to having just one control culture and requires further investigation. The presence of mucin (LW + M), on the other hand, triggered the intracellular CRISPR-Cas systems (as measured by spacer acquisition), and supported higher bacterial population densities. The extent of spacer acquisition in this condition was also positively affected by the presence of a competitor bacterium, highlighting that the surrounding microbiome may also play a synergistic role with mucin in determining defense strategies. The presence of mucin also led to more dramatic decline in phage titers, possibly through active removal of phages from the environment using the CRISPR-Cas systems^40^. In rich medium (Shieh), mucin also accelerated spacer acquisition during the first half of the experiment but with much lesser effect, while medium without mucin did not lead to spacer acquisition at all.

These results suggest that the role of CRISPR-Cas in *F. columnare* may be important specifically during colonization of the metazoan. In this setting, the fitness loss associated with surface modification is amplified by reduced bacterial virulence, as colony morphotype change leads to loss of virulence^33,40–42^ via mutations in the flavobacterial gliding motility genes associated with type 9 secretion system^35^. Similarly to previous findings^43^, most phage-exposed bacterial isolates collected in this experiment had mutations in *gld* or *spr* genes involved in the secretion of adhesins on the cell surface. These mutations also occurred in the LW + M treatment, indicating that CRISPR-Cas is not the sole resistance mechanism even in this condition. However, the phage concentration in our experimental system was likely higher than on natural fish surfaces, in which CRISPR-Cas mediated defense may be enough to carry the bacterium through the colonization process without compromising virulence through SM.

In general, bacteria isolated from all treatments throughout the 16-week experiment showed increased resistance to phage. Resistance was especially associated with the Rough colony morphotype. Surprisingly, the experimental conditions had an impact on the benefits of acquired CRISPR spacers. Expanded CRISPR arrays had a positive effect on bacterial growth in Rhizoid colonies (but negative effects on Rough colonies) in isolates originating from the LW + M treatment (Fig. 5a). However, in isolates from Shieh+M treatment, the result was opposite: Rough isolates had higher population density than Rhizoid with and without new spacers. Why specifically Rhizoid LW + M samples gained the most benefit from new spacers is unclear, but may reflect an altered bacterial metabolic state induced by the mucin signals indicating the presence of the fish host. If this phenomenon is widespread in bacterial pathogens, the finding may have important real-life implications for metazoan-based phage-bacterium interactions, which are not observed in standard laboratory cultures. Together, these results show that the benefit from additional spacers is affected by the environment where the acquisition happened and that spacers may even have a negative effect depending on the simultaneous surface modification. It is, however, worth noting that the growth experiment was performed in the nutrient-rich Shieh medium, which may downplay the activity of CRISPR-Cas as shown in the 16-week experiment.

Previous studies indicate that the high diversity of CRISPR spacers can drive phage populations into extinction^44^. However, our phage-bacterium system behaved differently. Despite the selection pressure by surface modifications and CRISPR-Cas defense, phage populations showed high genetic stability throughout the entire experiment, as clearly mutated phage genomes were found only in two replicates. In both cases, genetic change was observed in structural genes, indicating selection imposed by surface modification-based resistance, as seen also previously^45^. Both SM and CRISPR-driven changes in phage genomes have been observed also in the environment^31^, but despite the high prevalence of new CRISPR spacers in the LW + M treatment, no phage mutations were found in this treatment. We also did not find mutations in the pre-existing B245 spacer targets in any treatment. The lack of CRISPR evasion points to either inefficient interference, or an efficient degradation of phages with no leakage of mutated phages. The evolutionary potential of these phages may also be limited, as suggested by the slow emergence of mutations in natural samples over several years in this phage-bacterium system^31^. Interestingly, when we incubated phage V156 alone (no host) in LW + M conditions the phages went extinct already in the first sampling time (Supplementary Fig. 4), indicating that the viruses found in the host-containing cultures over the experiment were not leftovers from the initial inoculum but actively replicating progeny.

Diversity in bacterial communities has been shown to increase the benefits of CRISPR immunity in *P. aeruginosa*^29^. In our experiment, co-culturing *F. columnare* with another aquatic bacterium, *Aeromonas sp*, led to increased spacer acquisition in the competitive environment. New spacers were accumulated especially in the Rhizoid colony type, indicating a possible trade-off between surface resistance and competitive ability. While further exploring if the Rhizoid morphotype is a better competitor than Rough, we were surprised to discover that the presence of the Rhizoid morphotype decreased the growth of the competitor whereas Rough had no effect on the competitor. These dynamics suggest that the Rhizoid form of *F. columnare* actively suppresses the competitor through an unknown mechanism. The competitive advantage of the Rhizoid morphotype in a mucosal setting leaves the cell vulnerable to phage attacks, which may explain the high efficiency of spacer acquisition in this environment.

Our results support the view that phage defense strategies are influenced by ecological determinants, similar to previous studies showing the effects of nutrient and phage concentrations or the presence of competing species^1,3,29^. In our study system, the bacterium increased intracellular defenses in the presence of metazoan host signals, which may be an adaptive maneuver to maximize colonization by avoiding the predation by mucosal-associated phages. The necessity to avoid surface modification in mucosal settings may select for diversification of immune mechanisms, and partly explain why some CRISPR-Cas systems are enriched in pathogens^24^. It will be interesting to see if these results are generalizable to other phage-bacterium-metazoan systems. Furthermore, the roles of other intracellular systems besides CRISPR-Cas, such as restriction-modification, should be investigated. It is therefore clear that the phage-bacterium dynamics in the mucosa can be complex, and factors that are currently unknown can contribute to these interactions. From a practical viewpoint, understanding the interplay of bacterial virulence and phage defense during bacterial colonization of mucosal surfaces is crucial for the development of phage therapy, which specifically functions in this tripartite scenario.

## Methods

### Phage and bacteria

*Flavobacterium columnare* strain B245 was isolated from the same fish farm in Central Finland in 2009 as its phage V156^46^. Conventional culturing of B245 was made using Shieh medium^47^ without glucose. V156 stocks were produced by harvesting confluent soft-agar layers from double-agar plates, adding 4 ml of media, centrifuging (11.900 g, 10 minutes, Sorvall RC34 rotor) and filtering the supernatant through 0.22 µm filters. *Aeromonas sp*. B135 was isolated from a small natural brook in Central Finland (2008). B245, V156 and B135 are not present in biological collections but can be obtained from our personal collection by contacting the corresponding author. *E. coli* DSM613 strain was obtained from DSMZ GmbH (Braunschweig, Germany).

### Long-term culturing conditions and sampling

The effect of four different nutritional conditions on phage resistance mechanisms were studied: autoclaved lake water alone, lake water supplemented with 0.1% purified porcine mucin, 0.1x Shieh medium alone and 0.1x Shieh media supplemented with 0.1% purified porcine mucin. The lake water was collected from Lake Jyväsjärvi (Jyväskylä, Finland) on February 13^th^ 2018 and autoclaved. The water was analyzed by Eurofins Scientific and contained N: 790 µg L^−1^, P: 18 µg L^−1^ and Fe: 540 µg L^−1^. Shieh media was diluted in ultrapure sterile water. Autoclaved 2% w:v solution of purified porcine mucin (Sigma-Aldrich, catalog no. M1778) was used as stock for preparing the simulated mucosal cultures.

The initial inoculum in each culture was 5 × 10^4^ colony forming units (cfu) of *F. columnare* B245 and 5 × 10^3^ plaque forming units (pfu) of phage V156 (multiplicity of infection of 0.1). Each condition was tested in triplicates, in a final volume of five milliliters, and incubated at 26 degrees under 120 rpm. As non-infected controls, one culture of each condition was made with only B245 without phage. Every week after day zero, one milliliter of each culture was removed and replaced with one milliliter of the corresponding culturing media (autoclaved lake water or 0.1x Shieh, supplemented or not with 0.1% mucin). An overview of the experimental setup is shown in Fig. 1.

### Bacteria and phage titrations

Immediately after sampling, each sample was serially diluted and plated on Shieh-agar plates for analysis of bacterial population size. Chloroform (10% v:v) was added to the remaining sample to kill bacterial cells. Serial dilutions of the chloroform-treated supernatants were used for titrating phages with the double-agar layer method^48^ using the ancestral B245 as host. Bacteria and phage plates were incubated at room temperature for three days, followed by the enumeration of bacterial colonies and viral plaques. In the first experiment phages were titrated every week during the experiment, while bacteria were titrated in weeks 1 to 8, 10, 12 and 15. The total number of colonies used over this experiment is shown in Supplementary data 4. The competition experiment was sampled at days 7, 14, 32 and 56 for phage and bacteria titers. Twelve colonies per condition tested (four per replicate) were screened in each time point.

### Detecting the acquisition of new CRISPR spacers (both loci)

To investigate the spacer acquisition tendency of both colony types, we collected an equal number of Rough and Rhizoid colonies per sample (when possible) from the bacterial titration plates and transferred them to 50 microliters of Shieh on 96 well plates. Two microliters of each resuspended colony were used as template for PCR reactions designed to detect the insertion of new spacers on both CRISPR loci. Reactions were made with DreamTaq polymerase (Thermo Fisher), in 20 μL reactions containing 0.5 mM of DNTPs and 0.5 μM of primers designed to amplify each CRISPR loci: B245_C1_F1 (CTGTTTTGTTTCATTTGGTAAATCA) and CRISPR1R (CCCTAAAGCACCACAACCCA) or B185_CRISPR2_F1 (GGTCTAAATACAATTGCTCTTTGACATT) and B245_C2_R1 (GATGTAGAAATACTTAGCGACAATGTAG)^32^. Cycling conditions were 95 degrees for 3 minutes followed by 30 cycles of 95 degrees for 30 seconds, 60 degrees for 30 seconds, 72 degrees for one minute and a final extension step of 72 degrees for 15 minutes. PCR reactions were resolved in 2% agarose gels and the addition of spacers verified by the size of each amplicon.

### Bacterial growth characteristics

From the CRISPR-PCR colonies, a diverse set of bacterial isolates (aiming to maximize the number of different colony morphologies and CRISPR loci sizes) were chosen every week for further analysis (in total, from the 787 colonies tested by PCR, 133 were further analysed). Following the CRISPR-PCR, the remaining volume of resuspended colonies was transferred to 5 mL of Shieh media. After overnight growth (120 rpm, 26 degrees) the individual colonies/isolates were frozen at −80 degrees for future use and revived overnight for testing their immunity against the ancestral phage using Bioscreen C® (Growth curves Ltd, Helsinki, Finland). 1000 cfu mL^−1^ of each isolate were added to Bioscreen plates, in triplicates (200 microliters per well). Each isolate was tested in the presence and in the absence of the ancestral V156 phage (10^3^ pfu mL^−1^, MOI 1). Ancestral bacterial strain B245 was included on every plate as control, also in triplicates. Optical density measurements were made every 10 minutes for 4 days. Plates were kept without agitation at 27 degrees for the whole time. Minor differences between plates were accounted for by including the plate ID as a random effect in statistical models when appropriate. However, differences between plates were generally minimal (Supplementary Fig. 1). Testing the growth of bacterial isolates was not made for the competition experiment (see below).

### Genomic DNA extraction and sequencing

From the total of 133 bacterial isolates tested with Bioscreen, we chose 17 isolates (based on maximizing diversity in CRISPR spacers, morphology and phage resistance) for full genome sequencing. We also sequenced population-level phage DNA from week 16 samples and clonal bacterial isolates from different time points. For bacterial genomic DNA extraction, isolates were taken from the freezer and grown overnight. DNA of turbid cultures was extracted using the GeneJet Genomic DNA Purification Kit (Thermo Fisher). For phage DNA extraction, lysates from week 16 were used to infect B245. Confluent soft-agar bacterial lawns were collected, mixed with 4 ml of Shieh media, centrifuged (10.000 rpm, 10 minutes, Sorvall RC34) and filtered. Phage precipitation was made with ZnCl2 followed by removal of host DNA with nucleases^49^. After Protease K treatment, the material was mixed with Guanidine:Ethanol (1 part 6 M guanidine and 2 parts 99% ethanol, v.v.) and the extraction finished using the GeneJet Genomic DNA Purification Kit (Thermo Fisher). All samples were sequenced using Illumina 150PE BGISEQ platform at BGI Group. We were unable to obtain phage sequence data from the replicate *b* of the lake water with mucin condition due to technical problems.

#### Construction of the B245 reference genome

The ancestral B245 genome (accession number CP071008) was assembled from Illumina reads using Spades 3.14.1 (--isolate mode)^50^. The resulting 487 contigs were combined to a single contig relying on a previously compiled complete *F. columnare* genome FCO-F2 (accession number CP051861) as reference using RagOO 1.1^51^. The genome was annotated for the purpose of mutational analysis using dFast 1.2.3^52^.

#### Mutation analysis

We used Breseq 0.35.1^53^ to analyze mutations occurring in the phage and bacterial genomes. Since the phage samples represented mixed phage populations, Breseq was run in --polymorphism-prediction mode. Bacterial samples were run in default mode. As references we used the ancestral B245 bacterial genome or the previously published V156 phage genome (accession number KY979239.1)^31^. To reduce noise in phage mutation, we used a value of 0.05 for --polymorphism-bias-cutoff. During analysis of the phage genomes, we discovered a large number of identical polymorphisms in many samples. While this could be interpreted as a sign of convergent or parallel selection, it is more likely that these common mutations represent common sequencing artefacts or errors in the reference genome. As it is unlikely that these mutations would occur independently across several replicates in low frequency (especially with many mutations being synonymous), we discarded any identical mutations that co-occur in two or more samples, leaving only mutations with high confidence (however, the full variant dataset is available as a supplementary file). When investigating mutations in unknown putative phage genes, we used HHpred to predict protein function^54^. All bacterial and phage sequence data has been uploaded to SRA bioproject PRJNA842198; BioSample SAMN28649941 for bacteria and BioSample SAMN28649286 for phages.

### Competition experiment

To assess how competition affects spacer acquisition, we performed an experiment using only the lake water supplemented with 0.1% mucin condition (LW + M). The experimental setup was similar to the growth experiment described above, but besides adding 5 × 10^4^ cfu *F. columnare* B245 and 5 × 10^3^ pfu phage V156, we also included 5 × 10^4^ cfu of *Aeromonas sp*. B135 and of *E. coli* DSM613 as competitors. Cultures containing all three bacteria and V156, any combination of two bacteria and V156, and only *F. columnare* B245 host and V156 were made. Another control consisted of cultures containing only the V156 phage to follow its stability over time in the absence of its host. Each condition was tested in triplicates, in a final volume of five milliliters, and incubated at 25 degrees under 120 rpm. For this competition experiment we followed phage and bacterial titers, spacer acquisition in *F. columnare* and colony morphology.

To assess how morphotype (Rhizoid vs Rough) affects the competitive ability of *F. columnare* in the presence of mucin, we grew *Aeromonas sp*. together with either Rhizoid or Rough *F. columnare* in the LW + M condition without phage. As controls, each bacterial strain was also grown alone. Each condition was prepared in three replicates like the previous competition experiment and plated daily to calculate bacterial titers.

### Statistics/data analysis

All data analysis and statistic were done in R 3.5.3 using RStudio v_1.3.1093^55^. Model comparisons and inclusion of random effects were aided by Akaike’s information criterion (AIC) comparison when applicable^56^.

#### Effect of treatment of phage resistance

R library glmmTMB^57^ was used to analyze how the isolates differ from the ancestral bacterium in the presence or absence of phage. We used maximum OD reached during the follow-up growth experiment (ODMAX) as the response variable and the interaction between phage and treatment as the explanatory variables. We included replicate and bacterial ID as random effects and used a gaussian distribution as the response variable was normally distributed. The control samples were investigated using a linear model (lm in base-R) using the same interaction terms (the same random structure as in the phage-exposed samples could not be used due to the limited sample size in the control dataset). We also performed the analyses using time-to-ODMAX (the time it takes for a culture to reach its maximum OD) as the response variable but found this metric not to reflect the patterns that were shown by plaque assays and ODMAX measurements. We, therefore, omitted time-to-ODMAX from the final analysis.

#### Differences in titers of lake water with or without mucin

To reveal the effect of mucin on growth in lake water, we analyzed phage and bacterial titers from these conditions using a linear model in R. Titer was set as the response variable and treatment (LW or LW + M) as the explanatory variable. For phage titers, we only considered time points after 9 weeks, as the divergence between the two treatments started at this time (Supplementary Fig. 1a). As the bacterial titers diverged starting from week 1 (Supplementary Fig. 1b), we included all time points in the model. Similar analysis was not done for the Shieh and Shieh + mucin due to the large variation of replicate cultures in these conditions.

#### The effects of growth culture on spacer acquisition

The effects of mucin on spacer acquisition during the 16-week experiment was investigated using a generalized linear mixed model (glmmTMB package in R) with total number of spacers as the response variable and presence of mucin as the explanatory variable. Two tests were performed: one comparing LW with LW + M and one comparing LW with Shieh + M. (Shieh without mucin treatment was excluded from this analysis due to the minimal amount of acquired spacers). Treatment replicate ID was used as a random effect and negative binomial (nbinom2) as the distribution after AIC comparison.

#### The effect of spacers and morphology

To analyze the effects of spacers and morphology on ODMAX in the LW + M and Shieh+M treatments, we created a generalized linear mixed model (GLMM) using the R package glmmTMB. Both treatments were studied separately and categorized as having no new spacers or having one or more spacers. ODMAX was used as the response variable and predictors were [the presence of spacers] * [morphology]. As random effects, we included plate ID (the plate on which each sample was measured on) and replicate, which both increased model-fit considerably. A gamma distribution of the response variable was favored over other distributions with AIC difference >2.

#### The effect of phage on control samples

We examined if ODMAX of the control samples (grown in the four treatments without phage) in the growth experiment behaved similarly to the ancestral bacterium. We used a linear model (lm) in R with ODMAX as the response variable and treatment as the explanatory variable. The ancestral bacterium was used as an intercept against which all treatments were compared.

#### Effect of extraction time on ODMAX

Using a linear mixed model, we investigated the effect of extraction time on ODMAX. Including replicate as a random factor produced the best models according to AIC comparison. None of the samples showed a significant effect of isolation time on either response variable in the presence or absence of phage (Supplementary Fig. 3).

#### Effect of competition on F. columnare spacer acquisition

For the statistical analysis of the spacer acquisition/competition experiment with *Aeromonas sp*., we used a generalized linear model (glmmTMB package in R) with a total number of new spacers per colony as the response variable and condition (co-culture or not) as the explanatory variable. Replicate ID and sampling day were used as random effects. AIC model comparison of several distributions (including zero-inflated ones) indicated that a negative binomial distribution (nbinom2) fit the data best.

#### Effect of colony type on competitive ability

Comparison of bacterial titers was done using a generalized linear model (lme4 package in base R) using a Gamma distribution chosen by AIC model comparison. Bacterial titer of Aeromonas was used as the response variable and the condition as the response variable. The “*Aeromonas sp*. alone” was set as the intercept against which “*Aeromonas sp*. + *F. columnare* R” and “*Aeromonas sp*. + *F. columnare* Z” were compared. No random effects were used due to the small sample size.

### Reporting Summary

Further information on research design is available in the Nature Research Reporting Summary linked to this article.

## Supplementary information

Supplementary Information

Description of Additional Supplementary Files

Supplementary Data 1

Supplementary Data 2

Supplementary Data 3

Supplementary Data 4

Reporting Summary

## Data Availability

The datasets generated during the current study are available in Supplementary Information files, and in in the JYX repository (jyx.jyu.fi) provided by University of Jyväskylä, Finland: “10.17011/jyx/dataset/81288”^58^. Raw sequence data from bacterial isolates phage population sequences are available under the bioproject PRJNA842198. Source data are provided with this paper.

## Source data

Source Data

## Code availability

Code for data analysis and figure generation has been published in Zenodo^59^.
