# Supplementary material for: Mucin induces CRISPR-Cas defense in an opportunistic pathogen
Source: Nat Commun. 2022 Jun 25;13:3653. doi: 10.1038/s41467-022-31330-3 (PMC9233685; doi:10.1038/s41467-022-31330-3)
Supplement: Supplementary file 1 — Supplementary Information [file 41467_2022_31330_MOESM1_ESM.pdf]

Supplementary Information

Mucin induces CRISPR-Cas defense in an opportunistic pathogen

Almeida, Hoikkala, et al.

This file contains Supplementary Figures 1-4.

Supplementary figures

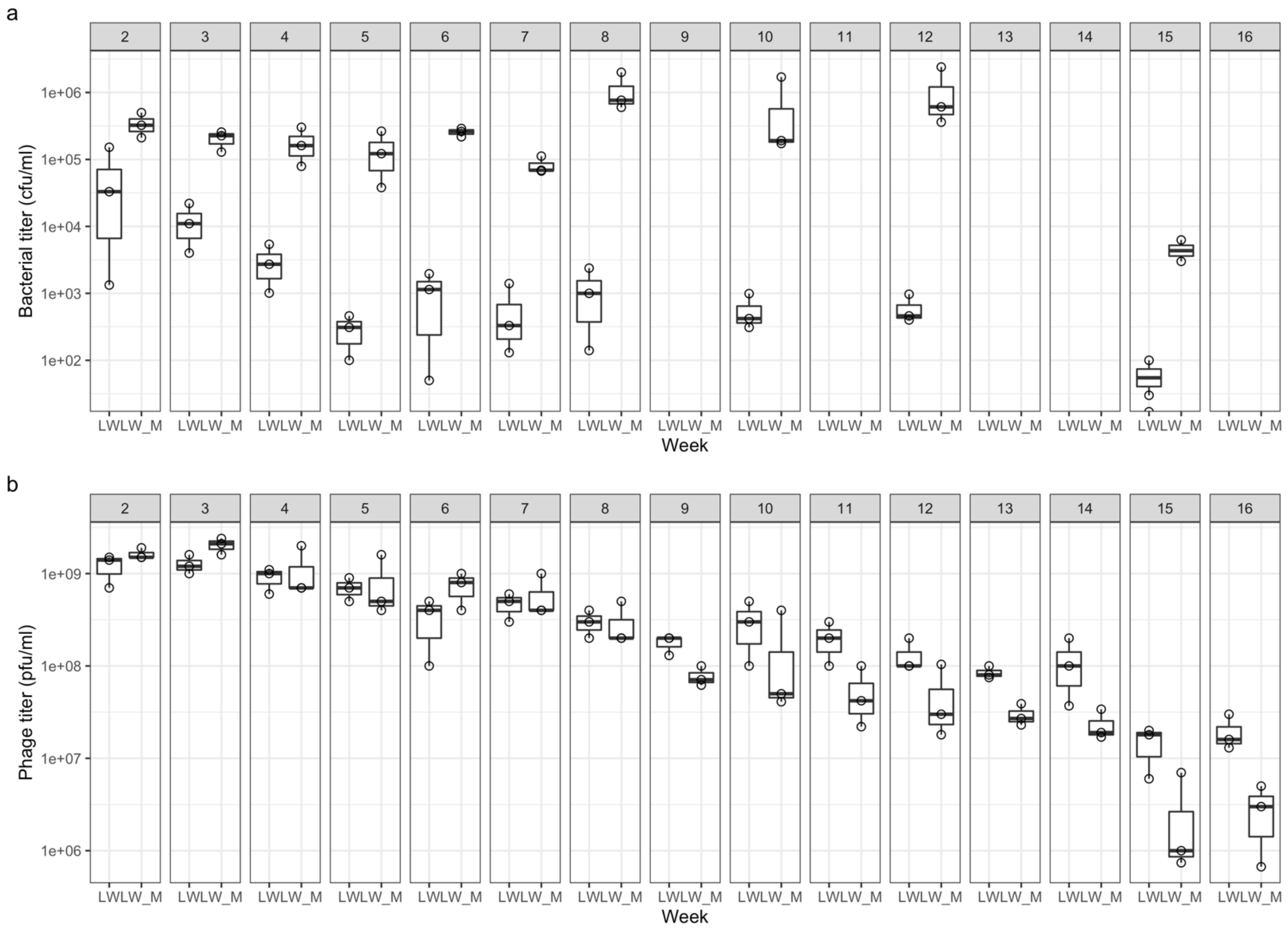

Supplementary Figure 1. Comparison of a) bacterial and b) phage titers in lake water (LW) and lake water supplemented with mucin (LW+M) during the 16-week experiment in the three replicate cultures (n=3, individual values are shown by dots, while the white box comprises first and third quartiles and the black line the median). Source data are provided as a Source Data file.

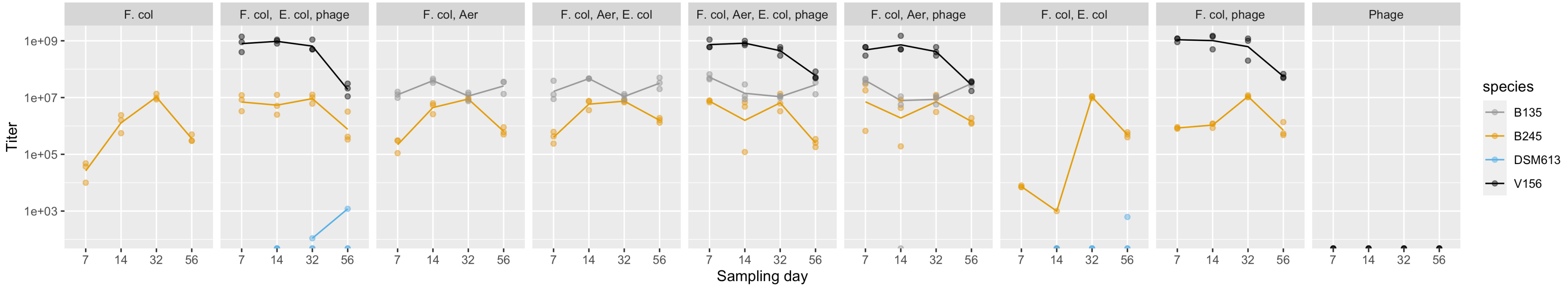

Supplementary Figure 2. Bacterial and phage titers from the competition experiments. Three bacterial species were competed in different combinations using three replicate cultures in the presence or absence of phage. B135 = *Aeromonas* sp., B245 = *F. columnare*, DSM613 = *E. coli*, V156 = phage. Source data are provided as a Source Data file.

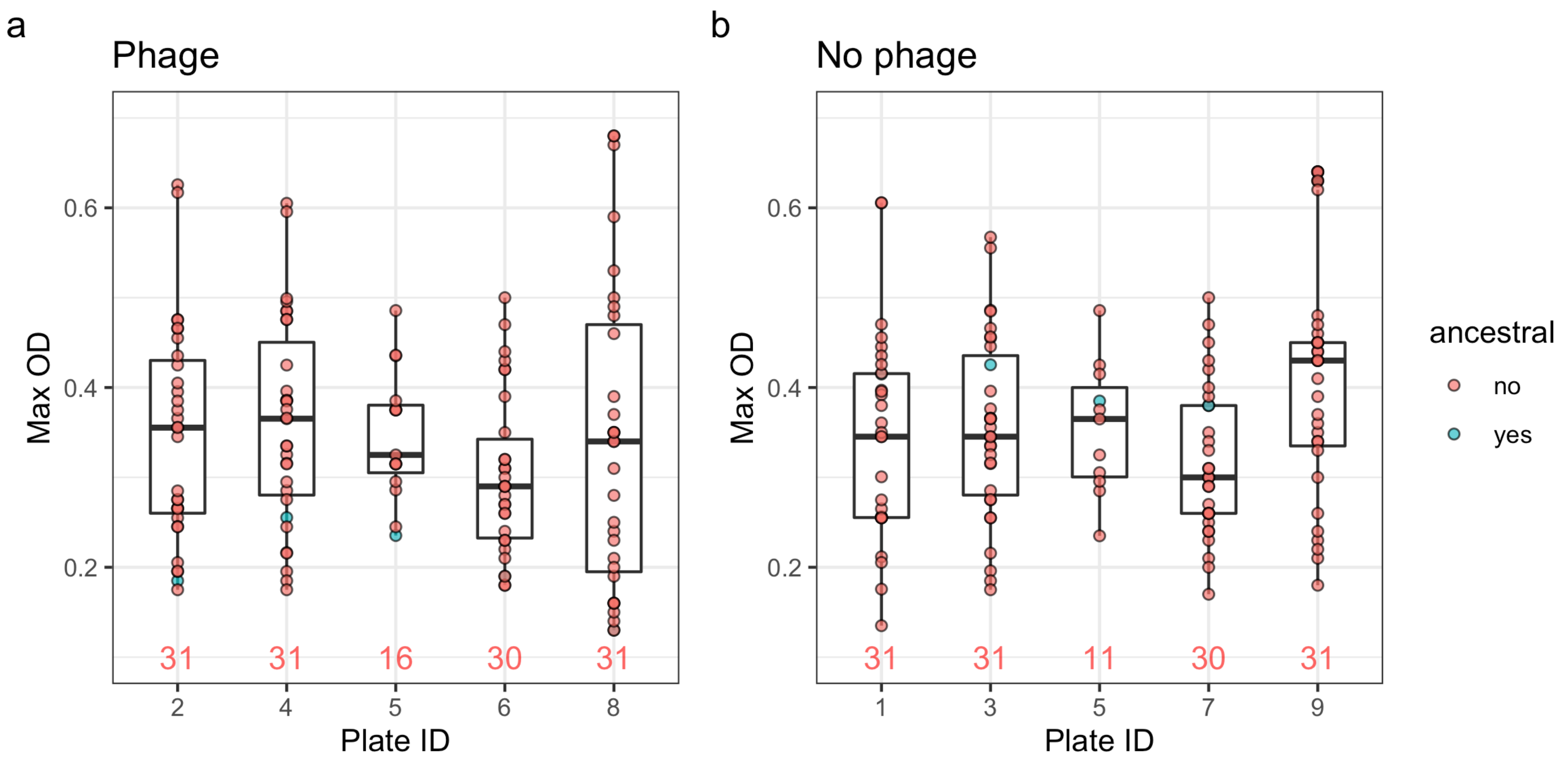

Supplementary Figure 3. Maximum OD reached by isolates in the a) presence or b) absence of the ancestral phage V156. The isolates shown here were extracted in various time points of the 16-week experiment, frozen and later reinfected with the phage while observing OD. The box plots capture the minimum and maximum values, 25th and 75th percentile (box) and the median (black horizontal line). "Plate ID" refers to BioScreen plate. A) Growth in the presence of phage B) Growth in the absence of phage. Source data are provided as a Source Data file.

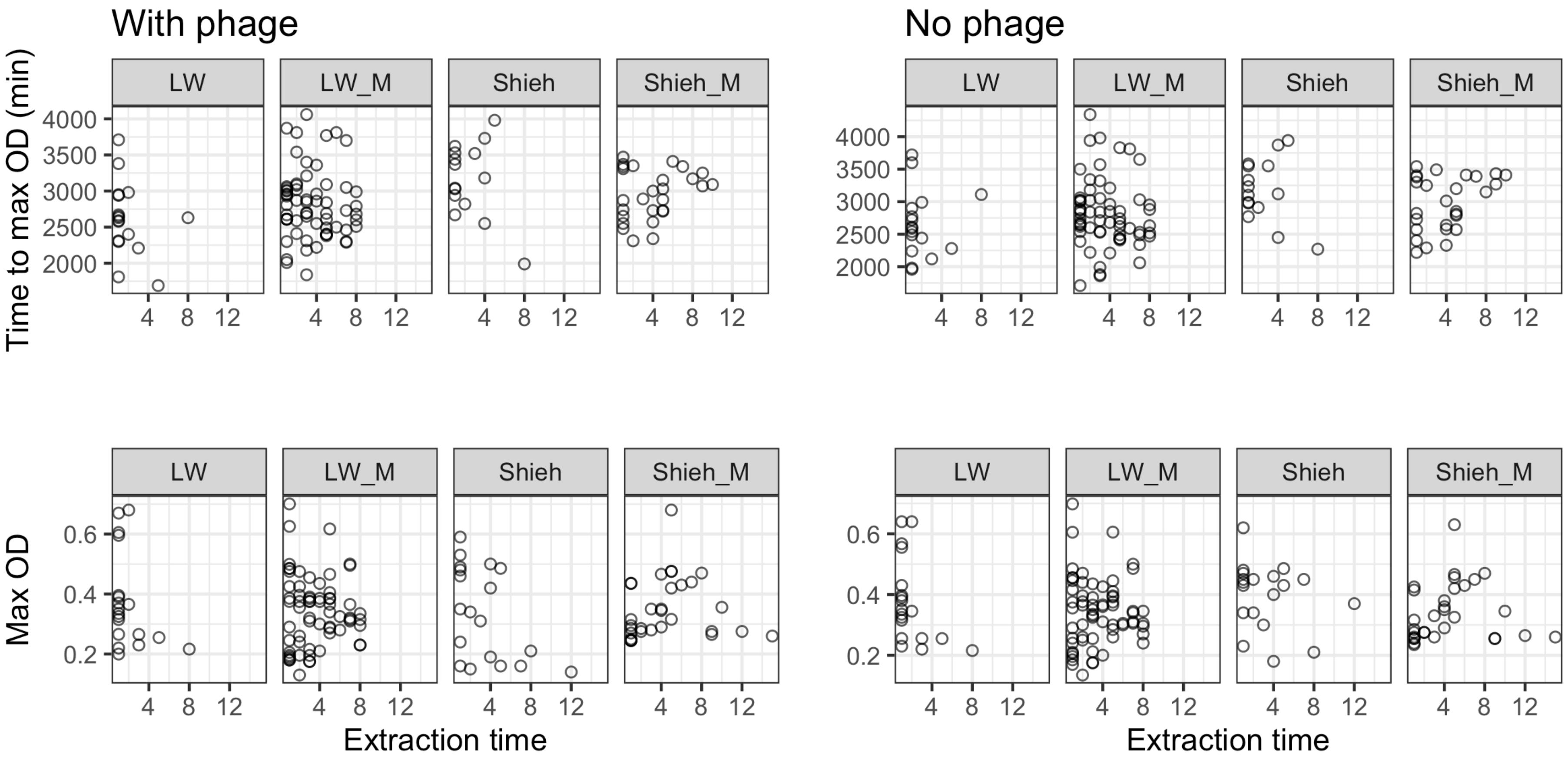

Supplementary Figure 4. The effect of extraction time point (weeks) of an isolate on their OD-max and time-to-OD-max in the presence or absence of phage. These samples were isolated at various time points during the 16-week experiment. Source data are provided as a Source Data file.
