# Supplementary material for: Mucin induces CRISPR-Cas defense in an opportunistic pathogen
Source: Nat Commun. 2022 Jun 25;13:3653. doi: 10.1038/s41467-022-31330-3 (PMC9233685; doi:10.1038/s41467-022-31330-3)
Supplement: Supplementary file 2 — Description of Additional Supplementary Files [file 41467_2022_31330_MOESM2_ESM.pdf]

Description of Additional Supplementary Files

File Name: Supplementary Data 1
Description: New spacers of a few Flavobacterium columnare CRISPR mutants isolated during the study, and phage susceptibility of selected isolates.

File Name: Supplementary Data 2
Description: Predicted mutations in phage genomes.

File Name: Supplementary Data 3
Description: : Predicted mutations in bacterial genomes.

File Name: Supplementary Data 4
Description: Number of colonies tested in each step of the co-evolution experiment
