# Supplementary material for: Mucin induces CRISPR-Cas defense in an opportunistic pathogen
Source: Nat Commun. 2022 Jun 25;13:3653. doi: 10.1038/s41467-022-31330-3 (PMC9233685; doi:10.1038/s41467-022-31330-3)
Supplement: Supplementary file 7 — Reporting Summary [file 41467_2022_31330_MOESM7_ESM.pdf]

nature portfolio

Corresponding author(s): Lotta-Riina Sundberg

Last updated by author(s): May 27, 2022

Reporting Summary

Nature Portfolio wishes to improve the reproducibility of the work that we publish. This form provides structure for consistency and transparency in reporting. For further information on Nature Portfolio policies, see our Editorial Policies and the Editorial Policy Checklist.

Statistics

For all statistical analyses, confirm that the following items are present in the figure legend, table legend, main text, or Methods section.

n/a	Confirmed	
☐	☒	The exact sample size (*n*) for each experimental group/condition, given as a discrete number and unit of measurement
☐	☒	A statement on whether measurements were taken from distinct samples or whether the same sample was measured repeatedly
☐	☒	The statistical test(s) used AND whether they are one- or two-sided<br>*Only common tests should be described solely by name; describe more complex techniques in the Methods section.*
☐	☒	A description of all covariates tested
☐	☒	A description of any assumptions or corrections, such as tests of normality and adjustment for multiple comparisons
☐	☒	A full description of the statistical parameters including central tendency (e.g. means) or other basic estimates (e.g. regression coefficient) AND variation (e.g. standard deviation) or associated estimates of uncertainty (e.g. confidence intervals)
☐	☒	For null hypothesis testing, the test statistic (e.g. *F*, *t*, *r*) with confidence intervals, effect sizes, degrees of freedom and *P* value noted<br>*Give P values as exact values whenever suitable.*
☒	☐	For Bayesian analysis, information on the choice of priors and Markov chain Monte Carlo settings
☐	☒	For hierarchical and complex designs, identification of the appropriate level for tests and full reporting of outcomes
☐	☒	Estimates of effect sizes (e.g. Cohen's *d*, Pearson's *r*), indicating how they were calculated

Our web collection on statistics for biologists contains articles on many of the points above.

Software and code

Policy information about availability of computer code

Data collection	No software was used to collect data in this study.
Data analysis	Rstudio 1.3.1093. Breseq 0.35.1. Spades 3.14.1. RagOO 1.1. dFast 1.2.3.

For manuscripts utilizing custom algorithms or software that are central to the research but not yet described in published literature, software must be made available to editors and reviewers. We strongly encourage code deposition in a community repository (e.g. GitHub). See the Nature Portfolio guidelines for submitting code & software for further information.

Data

Policy information about availability of data

All manuscripts must include a data availability statement. This statement should provide the following information, where applicable:

- Accession codes, unique identifiers, or web links for publicly available datasets
- A description of any restrictions on data availability
- For clinical datasets or third party data, please ensure that the statement adheres to our policy

The datasets generated during the current study are available in its Supplementary Information files, and in in the JYX repository (jyx.jyu.fi) provided by University of Jyväskylä, Finland: “https://doi.org/10.17011/jyx/dataset/81288". Raw sequence data from bacterial isolates and phage population sequences are available under the bioproject PRJNA842198: https://www.ncbi.nlm.nih.gov/sra/PRJNA842198.

Human research participants

Policy information about studies involving human research participants and Sex and Gender in Research.

Reporting on sex and gender	*Use the terms sex (biological attribute) and gender (shaped by social and cultural circumstances) carefully in order to avoid confusing both terms. Indicate if findings apply to only one sex or gender; describe whether sex and gender were considered in study design whether sex and/or gender was determined based on self-reporting or assigned and methods used. Provide in the source data disaggregated sex and gender data where this information has been collected, and consent has been obtained for sharing of individual-level data; provide overall numbers in this Reporting Summary. Please state if this information has not been collected. Report sex- and gender-based analyses where performed, justify reasons for lack of sex- and gender-based analysis.*
Population characteristics	*Describe the covariate-relevant population characteristics of the human research participants (e.g. age, genotypic information, past and current diagnosis and treatment categories). If you filled out the behavioural & social sciences study design questions and have nothing to add here, write "See above."*
Recruitment	*Describe how participants were recruited. Outline any potential self-selection bias or other biases that may be present and how these are likely to impact results.*
Ethics oversight	*Identify the organization(s) that approved the study protocol.*

Note that full information on the approval of the study protocol must also be provided in the manuscript.

Field-specific reporting

Please select the one below that is the best fit for your research. If you are not sure, read the appropriate sections before making your selection.

☒ Life sciences ☐ Behavioural & social sciences ☐ Ecological, evolutionary & environmental sciences

For a reference copy of the document with all sections, see nature.com/documents/nr-reporting-summary-flat.pdf

Life sciences study design

All studies must disclose on these points even when the disclosure is negative.

Sample size	No statistical methods were used to determine sample size. Experiments were performed in long term cultures made in triplicates, sampled repeatedly over time.<br><br>Our sampling size strategy is described in the manuscript and is as follows:<br>- Colony picking per sampling: To investigate the spacer acquisition tendency of both colony types, we collected an equal number of Rough and Rhizoid colonies per sample (when possible).<br>- Colonies tested after PCR: From the CRISPR-PCR colonies, a diverse set of bacterial isolates (aiming to maximize the number of different colony morphologies and CRISPR loci sizes) were chosen every week for further analysis.<br>- For sequencing: From the total of 133 bacterial isolates tested with Bioscreen, we chose 17 isolates (based on maximizing diversity in CRISPR spacers, morphology and phage resistance) for full genome sequencing.
Data exclusions	1) Phage sequencing was not possible for replicate b of the lake water with mucin condition, and this is stated in the text. 2) Our initial idea for the competition experiment was to have E. coli besides Aeromonas sp. and F. columnare. However, in our experimental conditions, E. coli did not survived long or remained at low levels during the experiment, and we decided to remove from the analysis every culture in which E. coli was present. So, in this experiment, we only analysed cultures containing F. columnare and Aeromonas sp. For clarity we mention the full experiment setup in the methods and then explain why E. coli containing cultures were excluded from the analysis and data presented.
Replication	Our repeated measurements of each culture allowed to follow the consistent values regarding bacterial and phage titers, colony morphology, and spacer acquisition. The replicates supported the trends seen. Whenever replicates varied significantly we mentioned in the text, such as for Shieh cultures in Figure 2.<br><br>Given the long term co-culturing nature of the experiments and the data obtained from repeated measurements of the same cultures over time, we did not replicated the experiments. Our major finding (spacer acquition in lake water plus mucin cultures) was confirmed in our competition experiment.
Randomization	No randomization was made because all experiments were in vitro and kept in the same conditions. When testing bacterial growth not all plates could be made at the same time. In this case plate ID was accounted in the models and no significant differences seen. This is shown in Supplementary Figure 1.
Blinding	No blinding was made. Samplings, data collection and data analysis were made in unbiased ways. The data obtained is well defined: colony morphotypes, band sizes in a gel, genomic information, and thus bias in data colllection is impossible.

Reporting for specific materials, systems and methods

We require information from authors about some types of materials, experimental systems and methods used in many studies. Here, indicate whether each material, system or method listed is relevant to your study. If you are not sure if a list item applies to your research, read the appropriate section before selecting a response.

Materials & experimental systems

n/a	Involved in the study
☒	☐ Antibodies
☒	☐ Eukaryotic cell lines
☒	☐ Palaeontology and archaeology
☒	☐ Animals and other organisms
☒	☐ Clinical data
☒	☐ Dual use research of concern

Methods

n/a	Involved in the study
☒	☐ ChIP-seq
☒	☐ Flow cytometry
☒	☐ MRI-based neuroimaging
